# Survival rate variation with different histological subtypes of poor prognostic male anal squamous cell carcinoma: a population-based study

**DOI:** 10.18632/oncotarget.20969

**Published:** 2017-09-16

**Authors:** Zihao Wan, Zhihao Huang, Vikash Vikash, Kelash Rai, Sindhu Vikash, Liaobin Chen, Jingfeng Li

**Affiliations:** ^1^ Department of Orthopaedic Surgery, Zhongnan Hospital of Wuhan University, Wuhan, Hubei Province, China; ^2^ Department of Colorectal and Anal Surgery, Zhongnan Hospital of Wuhan University, Wuhan, Hubei Province, China; ^3^ Department of Gastroenterology, Renmin Hospital of Wuhan University, Wuhan, Hubei Province, China; ^4^ Department of Medicine, Ziauddin Hospital, Karachi, Sindh, Pakistan; ^5^ Department of Medicine, Chandka Medical College, Shaheed Mohtarma Benazir Bhutto Medical University, Larkana, Sindh, Pakistan

**Keywords:** anus squamous cell carcinoma, male, tumor subtype, retrospective cohort study, SEER database

## Abstract

**Background and objective:**

The prognosis of male anal squamous cell carcinoma (MASCC) and female anal squamous cell carcinoma (FASCC) is variable. The influence of tumor subtype on the survival rate and gender is poorly known. Our study is the largest population-based study and aims to outline the difference in survival between MASCC and FASCC patients.

**Methods:**

A retrospective population-based study was performed to compare the disease-specific mortalities (DSMs) between genders related to the tumor subtypes. The Surveillance, Epidemiology, and End Results (SEER) program database was employed to obtain the data from January 1988 to December 2014.

**Results:**

A total of 4,516, (3,249 males and 1,267 females), patients with anal squamous cell carcinomas (ASCC) were investigated. The 5-year DSMs were 24.18% and 18.08% for men and women, respectively. The univariate analysis of the male basaloid squamous cell carcinoma (BSCC) and cloacogenic carcinoma (CC) patients demonstrated higher DSMs (P <0.001). Moreover, in the multivariate analysis, BSCC and CC were associated with soaring DSMs in male patients (P < 0.05).

**Conclusions:**

In the cohort of BSCC and CC patients, male patients demonstrated a considerable decrease in survival rate compared to females. A more precise classification of ASCC and individualized management for MASCC are warranted.

## INTRODUCTION

Anal cancer is a relatively rare neoplasm, accounting for approximately 1% of the gastrointestinal cancers. The incidence rate has increased steadily over the last four decades, particularly in males [1–3]. Anal squamous cell carcinoma (ASCC) is the most common subtype and constitutes 90% of all the anal cancers [4]. Literature reports the significant difference in the prognostic factors, survival outcome, and biological behaviors of tumor in male patients from females [4–6]. Soeberg et al. [6] report that the women with ASCC have a better prognosis the men. Over the past few years, studies related to epidemiology and histological features have suggested that MASCC should not be considered as a common disease and called for the need of customized diagnostic and therapeutic strategies.

The survival and mortality rate of MASCC patients have significantly improved, in spite of a limited number of conventional management or clinical protocols [7]. To the best of our knowledge, neither randomized control trials have been conducted to identify the optimal therapy for MASCC nor any consensus has been reached to address the factors responsible for the disparity in survival rate between men and women with ASCC.

The primary purpose of this study was to investigate the influence of the tumor subtype on the prognosis of MASCC, which might help to improve the diagnostic and therapeutic strategies of ASCC. Data from the National Cancer Institute’s Surveillance, Epidemiology, and End Results (SEER) program database was employed to determine the relationship of tumor subtype and prognosis by disease-specified mortality (DSM) for males and females suffering from ASCC. The National Cancer Institute does not require institutional review board approval for SEER studies because it is a publicly available and de-identified database.

## RESULTS

### Patient characteristics

A sum of 4,516 patients, (3,249 MASCC and 1,267 FASCC), were included in this study with a total duration of 27 years, extending from January 1988 to December 2014. This study encompasses four tumor subtypes 1: basaloid squamous cell carcinoma (BSCC), 2: cloacogenic carcinoma (CC), 3: nonkeratinizing large cell (NLC), and 4: verrucous carcinoma (VC). Table 1 listed the characters of participants and the values were statistically significant for each category (P < 0.05). The age of patients ranged from 17 to 104 years. The mean age (SD) at the time of diagnosis was 62.0(13.5) and 63.8(13.0) years for males and females, respectively. The Caucasian population accounted for the largest proportion followed by African-American in both men and women. Over 40% of patients with MASCC were single (never married) while 44.4% FASCC patients were married. The patients with MASCC had a high-grade tumor and was detected at an advanced stage. The mean tumor size (SD) was 36.4 (40.2) mm and 39.4 (47.0) mm in male and female, respectively (P > 0.05). Altogether, we identified four subtypes of ASCC, and the basaloid squamous cell carcinoma was the most common subtype accounting for 42.3% (males 39.1%, females 43.5%) of all cases. Patients undergoing surgical resection, radiotherapy and chemotherapy constituted the 36.1%, 82.3%, and 78.2% in MASCC group, respectively.

**Table 1:** Characteristics of patients with anus squamous cell carcinoma

Variables	Female	Male	P value
	N=3,249(%)	N=1,267(%)	
**Follow-up time, months**	69.5±63.1	61.2±59.4	<0.001
**Age at diagnosis, years**			<0.001
< 55	858 (26.4)	362 (28.6)	
55 - 65	882 (27.2)	348 (27.4)	
65 - 75	761 (23.4))	328 (25.9)	
≥ 75	748 (23.0)	229 (18.1)	
Mean (SD)	63.8 (13.0)	62.0 (13.5)	
Median	61.0	62.0	
**Race**			0.006
Caucasian	2894 (89.1)	1100 (86.8)	
Afro-American	234 (7.2)	127 (10.0)	
Other^a^	102 (3.1)	34 (2.7)	
Unknown	19 (0.6)	6 (0.5)	
**Marital status**			<0.001
Married	1441 (44.4)	485 (38.3)	
Unmarried^b^	1215 (37.4)	196 (15.5)	
Single (never married)	429 (13.2)	524 (41.4)	
Unknown	164 (5.0)	62 (4.9)	
**Primary site**			0.001
Anal canal	1272 (39.2)	462 (36.5)	
Cloacogenic zone	648 (19.9)	248 (19.6)	
Overlapping lesion	511 (15.7)	166 (13.1)	
Anus, unspecified	818 (25.2)	391 (30.9)	
**Tumor stage**			0.112
Localized	1669 (56.4)	645 (56.9)	
Regional	922 (31.1)	324 (28.6)	
Distant	371 (12.5)	165 (14.5)	
**Tumor grade**			0.006
Low	949 (29.2)	445 (35.1)	
High	2300 (70.8)	822 (64.9)	
**Tumor size**			0.475
Mean (SD)	36.4 (40.2)	39.4 (47.0)	
Median	30.0	30.0	
**Histological type**			<0.001
Basaloid squamous cell carcinoma	1414 (43.5)	495 (39.1)	
Cloacogenic carcinoma	1189 (36.6)	437 (34.5)	
Large cell, nonkeratinizing	585 (18.0)	203 (16.0)	
Verrucous carcinoma	61 (1.9)	132 (10.4)	
**Surgery**			<0.001
Yes	1173 (36.1)	551 (43.5)	
No	2060 (63.4)	710 (56.0)	
Unknown	16 (0.5)	6 (0.5)	
**Radiotherapy**			<0.001
Yes	2673 (82.3)	944 (74.5)	
No	532 (16.4)	303 (23.9)	
Unknown	44 (1.4)	20 (1.6)	
**Chemotherapy**			<0.001
Yes	2540 (78.2)	874 (69.0)	
No	709 (21.8)	393 (31.0)	

^a^Including Asian/Pacific Islander and American Indian/Alaska Native.

^b^Including separated, divorced and widowed.

### Influence of tumor subtype on DSM in MASCC and FASCC

**Figure 1:**
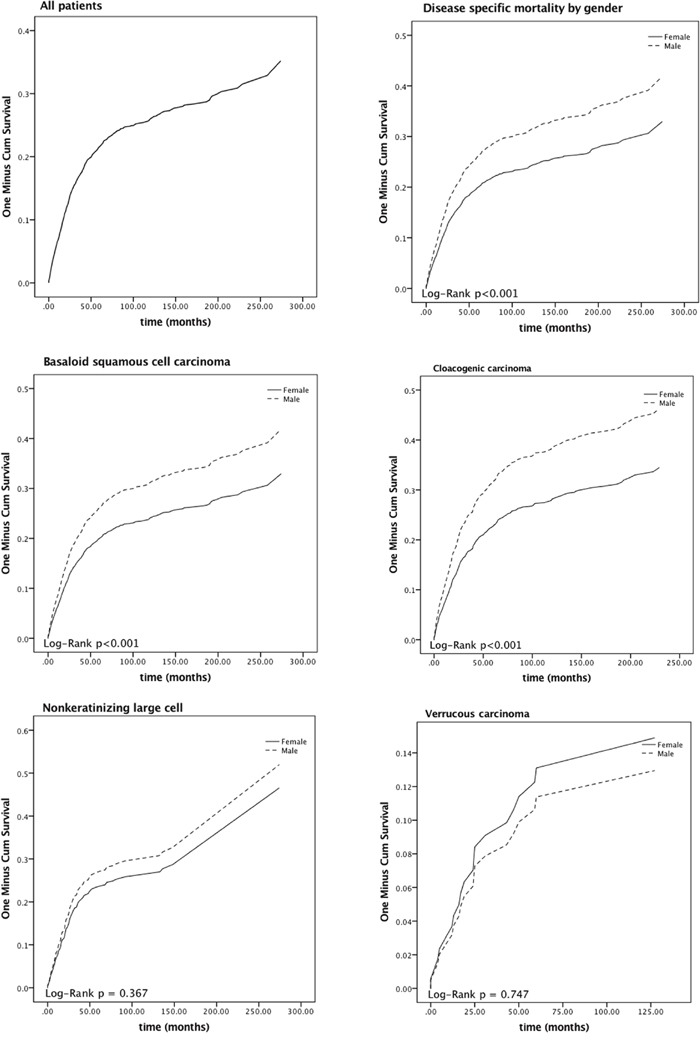
Disease-specific mortality curves of male paired with female anal squamous cell carcinoma patients

The Kaplan-Meier and the log-rank test were used to compute the DSMs and assess the difference, respectively. Figure 1 depicts the DSMs by gender and stratified by tumor subtypes. The 5-year DSMs were 24.18%, 18.08% for men and women, respectively. MASCC patients with BSCC and CC have higher DSMs compared to FASCC patients (P < 0.05).

Table 2 enlists the results of univariate and multivariate Cox proportional hazards analyses for the DSM. In the univariate analysis model, all the parameters, including age at the time of diagnosis, sex, race, marital status, primary site, tumor stage and grade, size, surgery, radiotherapy, and chemotherapy, were significantly associated with DSM (P < 0.05). In the Cox regression model, the age at the time of diagnosis, sex, race, primary site, tumor stage and grade, surgery, and chemotherapy were statistically significant (P < 0.05). The age at diagnosis (HR = 1.016 (1.005, 1.027)), male (HR =1.613 (1.227, 2.120)), Afro-American (HR = 1.793 (1.184, 2.716)), tumor stage ( regional, HR = 2.382 (1.779, 3.189); distant, HR = 6.815 (4.880, 9.518)), high tumor grade (HR = 1.442 (1.075, 1.934)), no surgery (HR = 1.310 (1.003, 1.712)), and no chemotherapy (HR = 1.971 (1.359, 2.859)).

**Table 2:** Cox proportional hazards regression model analyses of disease-specific mortality

Variables	Univariate analysis		Multivariate analysis^a^		
	HR(95%CI)	P value	HR(95%CI)	P value	
**Age at diagnosis, years**	1.018 (1.012,1.023)	<0.001	1.016 (1.005,1.027)	0.004	
**Sex**					
Female	Reference		Reference		
Male	1.358 (1.181,1.560)	<0.001	1.613 (1.227,2.120)	0.001	
**Race**					
Caucasian	Reference		Reference		
Afro-American	1.583 (1.283,1.953)	<0.001	1.793 (1.184,2.716)	0.006	
Other^b^	1.279 (0.908,1.804)	0.160	1.031 (0.525,2.022)	0.930	
**Marital status**					
Married	Reference		Reference		
Unmarried^c^	1.591 (1.366,1.853)	<0.001	1.295 (0.966,1.737)	0.084	
Single(never married)	1.387 (1.165,1.653)	<0.001	1.173 (0.841,1.635)	0.349	
**Primary site**					
Anal canal	Reference		Reference		
Cloacogenic zone	1.306 (1.102,1.547)	0.002	1.101 (0.792,1.532)	0.566	
Overlapping lesion	1.323 (1.092,1.603)	0.004	1.243 (0.888,1.740)	0.204	
Anus, unspecified	1.248 (0.741,1.057)	0.179	0.931 (0.665,1.305)	0.680	
**Tumor stage**					
Localized	Reference		Reference		
Regional	2.388 (2.024,2.817)	<0.001	2.382 (1.779,3.189)	<0.001	
Distant	7.234 (6.063,8.632)	<0.001	6.815 (4.880,9.518)	<0.001	
**Tumor grade**					
Low	Reference		Reference		
High	1.386 (1.125,1.706)	0.002	1.442 (1.075,1.934)	0.015	
**Tumor size**	1.003 (1.002,1.004)	<0.001	1.000 (0.998,1.001)	0.869	
**Surgery**					
Yes	Reference		Reference		
No	1.435 (1.248,1.649)	<0.001	1.310 (1.003,1.712)	0.047	
**Radiotherapy**					
Yes	Reference		Reference		
No	1.368 (1.166,1.606)	<0.001	0.987 (0.662,1.472)	0.951	
**Chemotherapy**					
Yes	Reference		Reference		
No	1.472 (1.275,1.699)	<0.001	1.971 (1.359,2.859)	<0.001	

^a^Adjusted for age at diagnosis, sex, race, marital status, site, tumor stage, tumor grade, size, surgery, radiotherapy and chemotherapy.

^b^Including Asian/Pacific Islander and American Indian/Alaska Native.

^c^Including separated, divorced and widowed.

Reference group for each model was “females.” cHR: crude hazard ratio; CI: confidence interval; aHR: adjusted hazard ratio (adjusted for age at diagnosis, sex, race, marital status, primary site, tumor stage, grade, tumor size and treatment modalities).

After the five years follow-up, the death proportion caused by ASCC in males and females are 24.18%, 18.08%, respectively. Table 3 established the distribution of DSM analysis for MASCC and FASCC subgroups. Based on tumor subtype, the univariate analysis of DSM displayed significant variation between MASCC and FASCC. Higher DSMs were observed in males with BSCC (5-year DSM: 24.31%) and CC (5-year DSM: 30.46%) compared to the female patients (log-rank P < 0.05). In the multivariate analysis, we also observed statistically significant differences between men and women in BSCC (HR = 2.089 (1.325, 3.295)) and CC (HR = 1.674 (1.218, 2.301)) subtypes. Moreover, these two tumor subtypes were recognized as the independent hazard indicators for poor survival in males (P < 0.05).

**Table 3:** Disease-specific mortality according to tumor subtypes between MASCC and FASCC

Tumor subtype	5-year DSM (%)		cHR (95% CI)	P value	aHR (95% CI)	P value
	Female	Male				
Basaloid squamous cell carcinoma	16.54	24.31	1.663(1.308,2.114)	<0.001	2.089(1.325,3.295)	0.002
Cloacogenic carcinoma	23.17	30.46	1.470(1.198,1.803)	<0.001	1.674(1.218,2.301)	0.001
Large cell, nonkeratinizing	24.01	25.75	1.171(0.830,1.650)	0.369	1.160(0.632,2.131)	0.632
Verrucous carcinoma	14.19	10.90	0.860(0.343,2.156)	0.748	0.026(0.001,4.244)	0.984

Reference group for each model was “female”. cHR: crude hazard ratio; CI: confidence interval; aHR: adjusted hazard ratio (adjusted for age at diagnosis, sex, race, marital status, site, tumor stage, tumor grade, size, surgery, radiotherapy and chemotherapy).

Reference group for each model was “females.” cHR: crude hazard ratio; CI: confidence interval; aHR: adjusted hazard ratio (adjusted for age at diagnosis, sex, race, marital status, primary site, tumor stage, grade, tumor size and treatment modalities).

## DISCUSSION

ASCC is rare tumor and is related to HPV, cigarette smoking, and immunosuppression [8–11]. ASCC and cervical carcinoma share several biological characteristics, such as the histopathologic appearance and association with HPV infection, but screening guidelines for ASCC are still unavailable [1, 12–14]. However, there is distinct variation in incidence rate and prognosis between MASCC and FASCC. In spite of some epidemiological investigations on MASCC, a limited number of the randomized controlled trial was carried out to demonstrate the biological features of MASCC and its treatment.

In our study, the mean (SD) ages at diagnosis were 57.9 (12.9) and 62.2(12.5) years for males and females, respectively. The result might reflect the prevalence of HIV infection although the data on HIV is missing in our analysis. The data reports that in the United States more than fifty percent of HIV patients are above the age of 50 years and estimated to be more than seventy percent by 2030 [15]. HIV is the most commonly transmitted by sexual route, and these patients are also prone to other sexually acquired infections, including anal HPV. In this population, HIV-associated immune deficiency and infection with high-risk HPV substantially increase the risk for the development of anal carcinoma [16].

Literature reports that the incidence of anal cancer in the homosexual male is higher than cervical cancer in the United States [17, 18]. To the best of our knowledge, the incidence rate of cervical cancer has declined during the past decades which could be attributed to the widespread implementation of the screening test, like Papanicolaou smear testing, while on the contrary, no routine test is being employed to screen for ACSS. Liszewski et al. [19] recommended the use of Papanicolaou smear testing for screening anal cancer, especially in the high-risk individuals.

A meta-analysis reports the detection of HPV infection in more than 80% of ASCC specimens [20]. The prevalence of HPV in the two major subtypes of ASCC, basaloid and large cell SCC, is similar [21]. In contrast to HPV-16 in cervical cancer, HPV-18 is prevalent in anal carcinoma [20, 22]. Tobacco smoking has been reported to increase the risk of infection with HIV, HPV, and also anal cancer [10, 23, 24], which is possibly due to carcinogenic and immunosuppressive effect [25].

Men who have sex with men, either HIV-positive or immunosuppressed, are at the increased risk of developing ASCC, and the incidence is progressing in this population [26–30]. Shiels et al. [31] declared that the prevalence of HIV in men was the prime cause of increased incidence of ASCC. However, HIV infection may not be a significant contributor for poor prognosis in MASCC patients compared to FASCC. In a retrospective analysis, Wieghard et al. [32] reported that there was no significant difference in recurrence rate and OS between HIV-positive (all male) and HIV-negative anal cancer patients.

Furthermore, we find out that over 40% of patients were single men, which may result in higher risk of developing ASCC. In male population, a history of being sexually active with a male was associated with anal carcinoma. Moreover, a case series proposed in cancer registry data that there is a higher incidence of anal cancer in single (never-married) than married men [33, 34] and also confirmed the increased risk for the development of ASCC in males in case-control studies [8–10, 35, 36]. All the studies on men who have sex with men pointed out the anal intercourse is the most important risk factor for ASCC development [9, 10, 35, 36].

From the genetic perspective, there is a low probability of Kirsten-ras (K-ras) and epidermal growth factor receptor (EGFR) mutations in ASCC, which raises the possibility for the therapeutic use of EGFR inhibitors such as Cetuximab in ASCC management [37]. The EGFR (HER-1 or ErbB1), a member of the tyrosine kinase receptor family, is well-known to stimulate tumor development via autocrine loop [38]. EGFR overexpression is related to poor prognosis, increased risk of tumor relapse, relative tolerance to treatment, and distant metastasis [39]. Literature reports that overexpression of EGFR in more than 90% of ASCC [37, 40–45]. However, there is not enough evidence of comparison of EGFR overexpression in between sexes, which requires further investigation.

We noticed a significant difference in between genders for basaloid squamous cell carcinoma (BSCC) subtype. In relation to that, literature reports the triple-negative immunophenotype, (estrogen receptor (-), progesterone receptor (-), and human epidermal growth factor receptor 2 (-)), and the expression of cytokeratin (CK), including CK5, CK14 or CK17, are considered as the gold standard for the diagnosis of BSCC [46–48]. Multiple studies have reported the poor of triple-negative carcinoma in comparison to other subtypes, especially in male patients [46, 49–51]. Foulkes and Turner et al. [52, 53] reported the association of BSCC carcinoma with BRCA1 germline, which suggests that anal BSCC carcinoma could be sensitive to platinum-based chemotherapy rather than mitomycin C because BRCA1 mutant carriers typically have somatic cell loss of the remaining BRCA1 allele and characterized by defects in double strand breaks DNA repair [54]. Reasonably, the difference in homologous gene mutations in tumor subtypes may contribute to the diagnosis and treatment of anal BSCC carcinoma.

Although the survival rate between genders varies significantly according to the histological subtypes, the confounding effect of other types of carcinomas was inevitable due to the restricted categorization. It was reported that about 30% of ASCC patients responded poor to chemoradiotherapy for unexplained reasons, and Graham et al. [55] discovered 10 in 37 samples primitively classified as BSCC were misdiagnosed, including basal cell carcinoma (n=6), melanoma (n=2), and neuroendocrine carcinoma (n=2) and a female predominance was also noticed in the final BSCC group. A precise classification system is in need despite the fact that WHO recommends using the term “squamous carcinoma” to cover all pathological types of ASCC.

Moreover, we noticed the decreased rate of survival for men with CC and NLC subtype. In a retrospective analysis, Serota et al. [56] report the frequent occurrence of CC in women, while men were reported with more glandular variations of CC, early metastasis to lymphoid follicles and other organs and decreased survival compared to women. It is quite challenging to distinguish CC from other ASCCs owing to same clinical behavior, and the standard of care [57, 58]. Unfortunately, in recent years, no consistent study compared the prognosis of patients with NLC to the other ASCCs. However, we observed an apparent difference in survival in between genders in the NLC subgroup.

With regards to the limitations of our study, we were unable to assess molecular indicators, such as EGFR which could lead to the early diagnosis and anticipate the prognosis. Moreover, the conclusions derived from retrospective studies were of a low methodological grade in contrast to the randomized controlled trials. Furthermore, due to the anonymous rule in the SEER program, we were unable to contact the patients for detailed information. Finally, information regarding comorbidities and tumor relapse is not documented in the database.

## MATERIALS AND METHODS

### Data source and study design

We used the SEER program database (Incidence - SEER 18 Regs Research Data + Hurricane Katrina Impacted Louisiana Cases, Nov 2016 Sub, 1988-2014 varying) to acquire data of patients diagnosed with ASCC from January 1988 to December 2014. Histologic International Classification of Diseases (ICD) codes, third edition (ICD-0-3) were undertaken to identify squamous cell carcinoma (8050/3-8053/3, 8070/3-8076/3, 8083/3, 8084/3, 8123/3, 8124/3). Anatomic codes (C21.0-C21.2, C21.8) were employed to screen for neoplasm originating in the anus. The data was extracted from the SEER database for analysis, including age at diagnosis, sex, marital status, race, primary site, histological type, tumor size, tumor stage, differentiation, treatment modality, survival status, the cause of death, and survival months. Cases without details on pathological types, samples less than 90 cases, patients diagnosed on autopsy, and the patients who were not confirmed microscopically were excluded from our study. We divided the differentiations into two categories (well-differentiated and moderately differentiated histologic features were defined as low grade, and poorly differentiated and undifferentiated characters were asserted as a high degree). The population was allocated into four groups for the subgroup analysis on the basis of histological types.

### Statistical analysis

The primary result of our investigation was disease-specific mortality (DSM). All the variables were statistically characterized. DSW was computed by the Kaplan-Meier method, and the log-rank test was applied to examine the difference. We figured hazard ratios (HRs) and the 95% confidence interval (CI) using the Cox proportional hazards model. The multivariate Cox proportional hazards model was performed to calculate the mortality-association risk factors in patients with ASCC after adjusting for other variables. We used multiple imputation by Monte Carlo Markov Chain method to estimate missing values. SEER^*^Stat version 8.3.4 (IMS Inc, USA) was used to extract data. SPSS version 23 (IBM Corp, USA) was used to accomplish all the statistical analyses. The R statistical software version 3.3.1 ( www.r-project.org) was employed to perform multiple-imputation. Differences between the groups were considered statistically significant if the P values were less than 0.05.

### Ethical statements

The National Cancer Institute and the Ethics Review Board of Zhongnan Hospital of Wuhan University found that the institutional review board approval is not required for the SEER study as it utilizes the unidentified public-use database.

## CONCLUSION

To sum up, the SEER program database is of significant advantage despite these restrictions and provides feasibility for researching huge population with rare cancers. We report noticeable differences between MASCC and FASCC DSM in various subtypes. Further studies are required to for the precise classification of ASCC and individualized management of MASCC.

## Abbreviations

MASCC: male anal squamous cell carcinoma
FASCC: female anal squamous cell carcinoma
DSM: disease-specific mortality
ASCC: anal squamous cell carcinomas
BSCC: basal squamous cell carcinoma
CC: cloacogenic carcinoma
VC: verrucous carcinoma
NLC: nonkeratinizing large cell
